# Diabetes Trajectory and Pancreatic Cancer Detection in Patients with Diabetes: A Nationwide Survey Through Japan Diabetes Society-Certified Facilities

**DOI:** 10.3390/cancers18182995

**Published:** 2026-09-16

**Authors:** Atsushi Masamune, Yuichi Tanaka, Yu Tanaka, Tetsuya Takikawa, Yasumasa Sekino, Ryotaro Matsumoto, Yoichiro Asai, Hideki Katagiri, Kohjiro Ueki, Kazuhiro Kikuta

**Affiliations:** 1 Division of Gastroenterology, Tohoku University Graduate School of Medicine, Sendai 980-8574, Japan; 2 Department of Gastrointestinal Aging Control, Healthspan Research Center, Tohoku University, Sendai 980-8575, Japan; 3 Department of Diabetes, Metabolism and Endocrinology, Tohoku University Graduate School of Medicine, Sendai 980-8574, Japan; 4 SiRIUS Institute of Medical Research, Tohoku University, Sendai 980-8575, Japan; 5 Diabetes Research Center, National Institute of Global Health and Medicine, Japan Institute for Health Security, Tokyo 162-8655, Japan

**Keywords:** diabetes mellitus, diabetes trajectory, ENDPAC, new-onset diabetes, pancreatic cancer, pancreatic ductal adenocarcinoma

## Abstract

**Background/Objectives**: Diabetes mellitus (DM) and pancreatic cancer (PC) have a bidirectional relationship. Although new-onset DM (NODM) is recognized as a potential clue to PC, worsening glycemic control in long-standing DM (LSDM) may also precede PC diagnosis. We aimed to characterize patterns of PC detection in specialist diabetes practice and to examine the association between diabetes-related diagnostic triggers and stage at diagnosis. **Methods**: This retrospective, nationwide, two-stage survey included patients with PC and DM treated at Japan Diabetes Society-certified educational facilities between 2017 and 2021. NODM and LSDM were defined as diabetes diagnosed within and more than 3 years before PC diagnosis, respectively. **Results**: Among 2291 patients with PC, 549 (24.0%) had NODM and 1742 (76.0%) had LSDM. Compared with symptom-based diagnosis, diabetes-related detection was associated with a higher proportion of stage 0/IA disease (11.0% vs. 3.9%) and a lower proportion of stage IV disease (26.1% vs. 49.5%). Diabetes-related detection was independently associated with stage 0/IA disease, with an adjusted odds ratio of 2.87 (95% confidence interval, 1.72–4.81; *p* < 0.001). Among patients with worsening LSDM, 57.2% were diagnosed with PC within 3 months and 79.4% within 6 months. Among those with available longitudinal body-weight data, 66.9% experienced weight loss of at least 2.5% compared with 1 year before PC diagnosis. **Conclusions**: Potential clues to PC in diabetes practice extend beyond NODM to deterioration of pre-existing diabetes, with weight loss frequently observed among patients with available longitudinal data. Prospective studies with appropriate control groups are needed to determine which diabetes trajectories can reliably identify patients who warrant targeted pancreatic evaluation.

## Simple Summary

Pancreatic cancer is difficult to detect at an early stage, and changes in glycemic control may occur before the cancer is diagnosed. Previous research has mainly focused on new-onset diabetes as a possible clue to pancreatic cancer. We conducted a nationwide survey through Japan Diabetes Society-certified facilities to characterize how pancreatic cancer was diagnosed among patients receiving specialist diabetes care and to examine diabetes-related changes preceding diagnosis. Among 2291 patients with pancreatic cancer and diabetes, approximately three-quarters had pre-existing rather than newly diagnosed diabetes. Worsening diabetes preceded pancreatic cancer diagnosis in many patients, and cancers detected because of diabetes-related changes were more often diagnosed at an earlier stage than those detected after the development of symptoms. These findings suggest that attention should be paid not only to new-onset diabetes but also to unexpected worsening of pre-existing diabetes, particularly when accompanied by weight loss. Further prospective studies are needed to determine whether these changes can reliably identify patients who should undergo pancreatic evaluation.

## 1. Introduction

Pancreatic cancer (PC), predominantly pancreatic ductal adenocarcinoma, remains one of the most lethal malignancies worldwide [1,2]. In 2022, approximately 510,600 new cases and 467,000 deaths from PC were estimated globally [3]. In Japan, PC was the third leading cause of cancer-related death in 2024, accounting for 41,235 deaths, and the 5-year relative survival rate for patients diagnosed in 2018 was only 13.2% [4,5]. The poor prognosis is largely attributable to the difficulty of detecting PC at an early, resectable stage. Because population-based screening of asymptomatic individuals is not currently recommended [6], identifying clinical situations that provide opportunities for the detection of occult PC is an important strategy for improving outcomes [1,2,7,8].

Diabetes mellitus (DM) is a risk factor for PC and has a well-established bidirectional relationship with the disease [9,10,11,12,13]. Long-standing DM (LSDM) is associated with an increased risk of PC, whereas PC itself can cause new-onset or worsening DM through tumor-related metabolic alterations [9,10,11,12]. A meta-analysis of 88 studies demonstrated that DM was associated with a relative risk of 1.97 for developing PC [14]. Notably, this risk was inversely associated with the duration of DM: 6.69 for less than 1 year, 1.86 for 1–4 years, 1.72 for 5–9 years, and 1.36 for 10 years or more, suggesting that new-onset DM (NODM) may represent an early manifestation of PC. NODM, particularly in older individuals, is therefore recognized as a potential early manifestation of occult PC [15,16,17,18,19]. However, DM is common, and only a small proportion of patients with DM subsequently develop PC; thus, DM alone has limited specificity for cancer detection.

Several studies have shown that weight loss and worsening glycemic control preceding PC diagnosis are more pronounced among patients who subsequently develop PC [18,20,21,22]. In particular, longitudinal increases in glucose and glycated hemoglobin A1c (HbA1c), together with preceding weight loss, have been associated with PC development among patients with NODM. These observations led to the development of the Enriching New-Onset Diabetes for Pancreatic Cancer (ENDPAC) model, which combines age at diabetes onset, change in body weight, and change in blood glucose category to identify patients at increased risk of PC [18]. More recent prospective and genetic studies, including those incorporating machine-learning approaches, have further supported the potential value of clinical and biological features for risk stratification in NODM [23,24,25,26,27]. These findings suggest that the longitudinal trajectory of diabetes may provide information about occult PC beyond the presence of diabetes itself [7,9,28,29]. However, this concept has been studied predominantly in the context of NODM. Less is known about how PC is actually detected among patients with established diabetes receiving specialist care, particularly whether unexpected deterioration in long-standing diabetes may provide a clinically relevant opportunity for diagnosis before cancer-related symptoms emerge.

We therefore aimed to characterize PC detection in specialist diabetes practice, with particular focus on diabetes-related changes preceding PC diagnosis and their association with stage at diagnosis.

## 2. Materials and Methods

### 2.1. Study Design and Study Population

This was a retrospective, multicenter, two-stage nationwide survey conducted with the support of the Japan Diabetes Society (JDS). The first-stage survey was designed to estimate the frequency of PC among patients with DM receiving care at JDS-certified educational facilities. The second-stage survey was designed to collect detailed demographic and clinical information on individual patients with PC.

In July 2022, questionnaires were sent to all 856 educational facilities certified by the JDS. Each facility was asked to report the average annual number of patients with DM who visited the institution between January 2017 and December 2021 and the total number diagnosed with PC among them during the same period. PC was not specifically defined as pancreatic ductal adenocarcinoma. For each facility, the reported frequency of PC per 1000 patients with DM was calculated as follows: (total number of patients diagnosed with PC during 2017–2021/5)/average annual number of patients with DM receiving care during the same period × 1000. Facilities reporting one or more patients with PC were subsequently invited to participate in the second-stage survey.

In January 2023, a second questionnaire was sent to participating facilities to obtain demographic and clinical information on individual patients with PC. Among patients for whom relevant information was available, body-weight change was calculated as [(body weight at PC diagnosis − body weight 1 year before PC diagnosis)/body weight 1 year before PC diagnosis] × 100; thus, this analysis represented change between these two time points rather than maximum weight loss during the preceding year. HbA1c change was calculated as the HbA1c value at PC diagnosis minus that 1 year before PC diagnosis and was expressed in percentage points.

The interval between worsening of pre-existing DM and PC diagnosis was also recorded. Worsening of pre-existing DM was based on the clinical assessment recorded at each participating facility and was not defined by a prespecified quantitative threshold for HbA1c, fasting glucose, glucose variability, or treatment escalation. Data collection was conducted online through March 2024. Clinical stage was determined according to the Japanese classification of pancreatic carcinoma 8th edition [30]. Stage 0 was defined as high-grade pancreatic intraepithelial neoplasia or pancreatic carcinoma in situ without regional lymph node involvement or distant metastasis. Stage IA was defined as a tumor limited to the pancreas, with a size of 20 mm or less in the largest diameter, without regional lymph node involvement or distant metastasis. In this study, stages 0 and IA were considered early-stage PC, consistent with previous studies in which these stages have been regarded as key targets for early detection because of their favorable long-term prognosis [31,32].

### 2.2. Definition of NODM and LSDM

NODM was defined as diabetes diagnosed within 3 years before PC diagnosis, whereas LSDM was defined as diabetes diagnosed more than 3 years before PC diagnosis. This definition was selected to capture the period during which diabetes is most likely to represent a manifestation of occult PC rather than a conventional long-standing metabolic risk factor [18]. Diabetes-related detection was defined as PC diagnosed in association with either NODM or worsening glycemic control in patients with LSDM. Diagnostic triggers were categorized according to the primary clinical event that led to the diagnosis of PC.

### 2.3. Statistical Analysis

Continuous variables are presented as mean ± standard deviation (SD) or median with interquartile range (IQR). Continuous variables were compared using the unpaired Student’s t-test or the Mann–Whitney U test, as appropriate. Categorical variables were compared using the chi-square test or Fisher’s exact test, as appropriate. Missing data were not imputed. Analyses were performed using available cases for each variable, and the number of patients with available data is reported where appropriate.

To examine whether the association between diagnostic trigger and disease stage was independent of potential confounding factors, we performed a multivariable binary logistic regression analysis. The primary explanatory variable was diagnostic trigger (diabetes-related vs. symptom-based detection). Covariates were selected a priori based on established PC risk factors and clinically relevant demographic and disease-related characteristics that could potentially confound the association between the diagnostic triggers and stage at diagnosis [33]. The multivariable model included age, sex, body mass index (BMI), history of pancreatic disease, family history of pancreatic cancer, smoking history, and alcohol drinking history. Tumor size was not included because it is closely related to the stage outcome; tumor location may similarly influence symptom development and diagnostic trigger and was not included as a confounder.

Adjusted odds ratios (ORs) with 95% confidence intervals (CIs) were estimated, and *p* values were assessed using likelihood ratio tests. As a sensitivity analysis, a mixed-effects logistic regression model with institution included as a random intercept was performed to account for potential clustering of patients within institutions.

All statistical tests were two-sided, and *p* < 0.05 was considered statistically significant. Statistical analyses were performed using SPSS Statistics version 20.0 (SPSS Inc., Chicago, IL, USA) and JMP Student Edition 19.1.2 (SAS Institute Inc., Cary, NC, USA).

### 2.4. Ethics

This study was approved by the Ethics Committee of Tohoku University Graduate School of Medicine (2022-1-755 and 2022-1-756). Because of the retrospective nature of the study, the requirement for individual informed consent was waived. Information regarding the study was made publicly available on the institutional website, and patients were provided with the opportunity to opt out of participation.

## 3. Results

### 3.1. Frequency of PC Among Patients with DM

**Figure 1:**
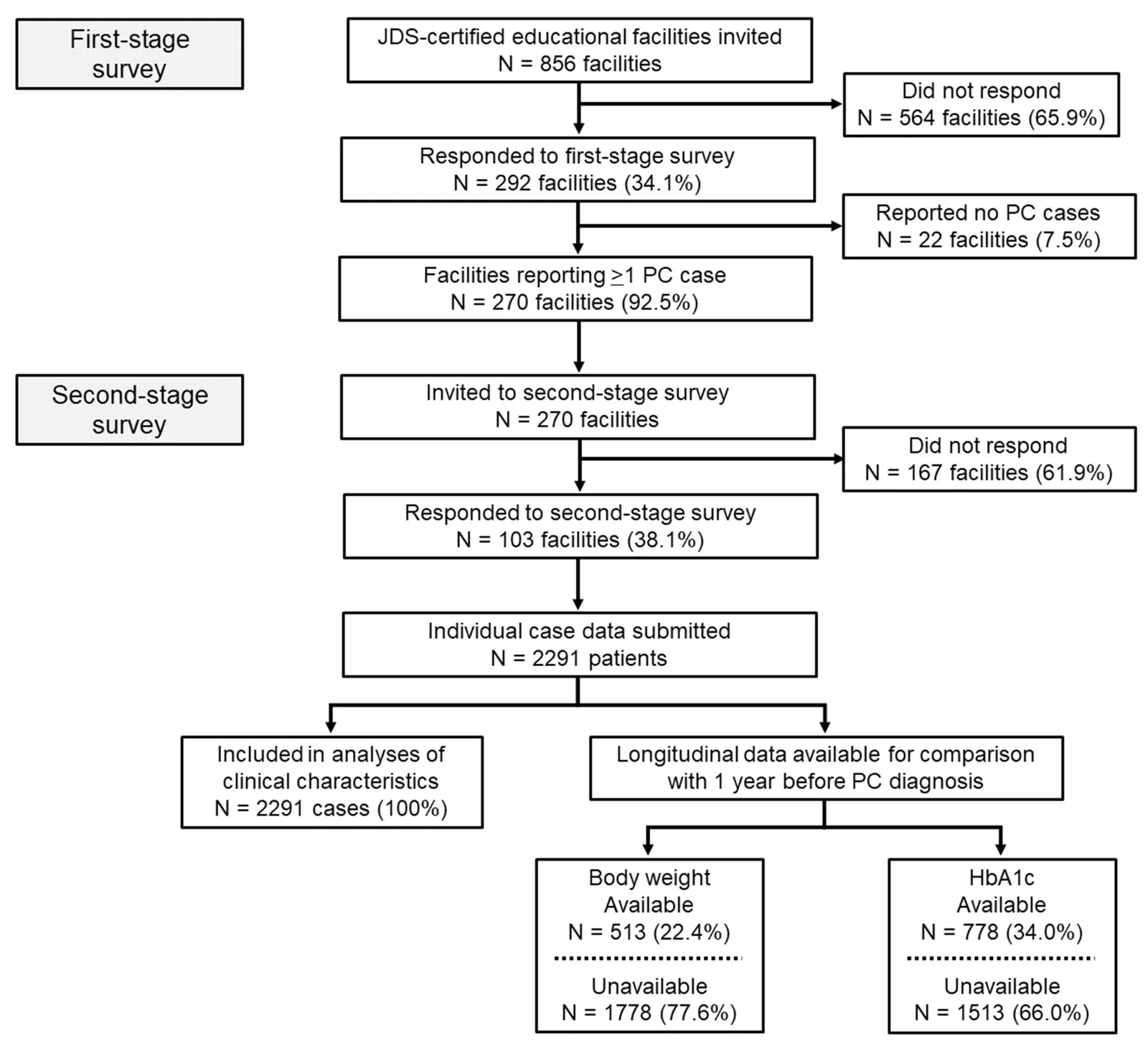
Flowchart of the two-stage survey. JDS, Japan Diabetes Society; PC, pancreatic cancer.

The flowchart of the two-stage survey is shown in Figure 1. In the first-stage survey, 292 of 856 facilities responded, corresponding to a response rate of 34.1%. The median number of patients with DM treated at participating facilities per year was 1590.5 (IQR, 925.3–2480). The median facility-level frequency of PC was 2.0 (IQR, 0.9–4.9) per 1000 patients with DM. This descriptive measure should not be interpreted as a standard prevalence or incidence estimate.

### 3.2. Clinical Characteristics of Patients with PC

The second-stage survey yielded clinical information for 2291 patients with PC from 103 facilities (Table 1). The male-to-female ratio was 1.67:1. The mean BMI was 22.6 kg/m^2^. A history of pancreatic disease was present in 253 patients (11.4%), whereas a family history of PC was reported in 138 patients (8.1%). Alcohol consumption was reported in 1000 patients (47.2%), and 1155 patients (53.4%) had a history of cigarette smoking.

**Figure 2:**
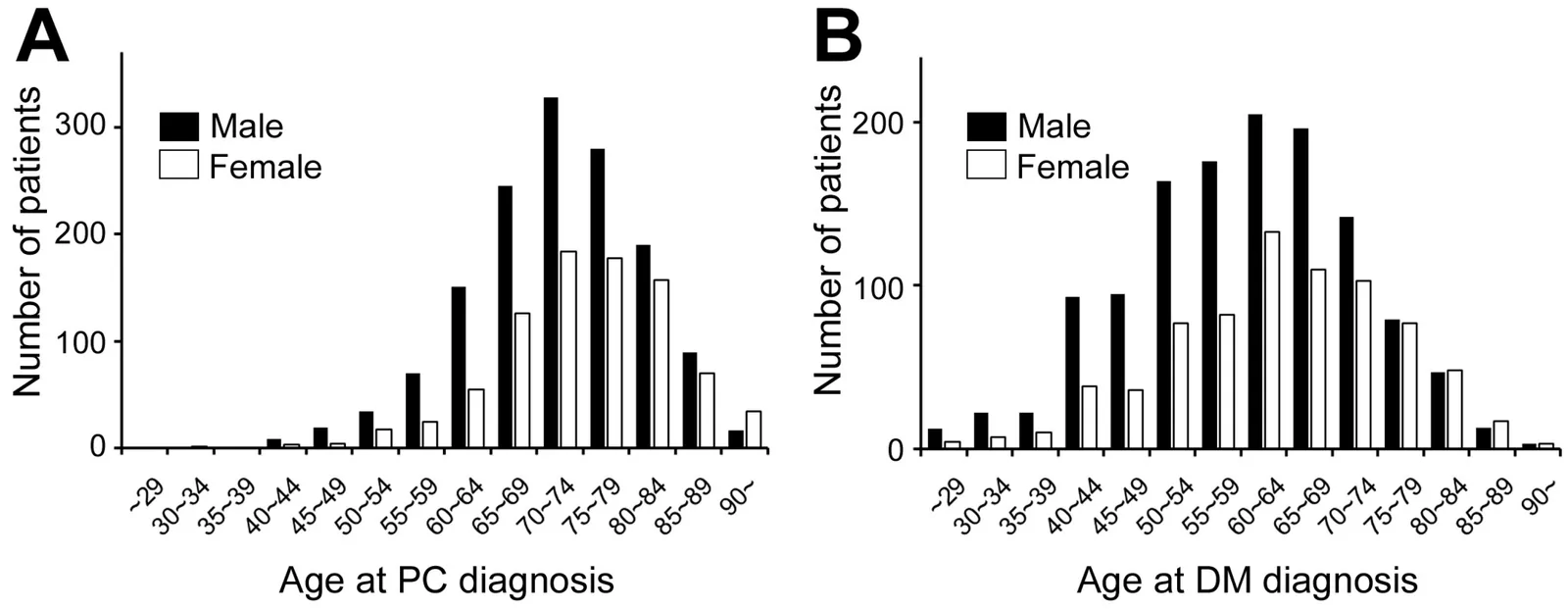
Distribution of age at PC and DM diagnosis stratified by sex. (**A**) Age at PC diagnosis was available for 1430 male and 852 female patients. (**B**) Age at DM diagnosis was available for 1269 male and 745 female patients.

The mean age at DM diagnosis was 60.9 years, and the mean age at PC diagnosis was 72.8 years (Figure 2). The peak age group for PC diagnosis was 70–74 years in both men and women. Among 2011 patients with available information, the mean interval between DM and PC diagnosis was 11.5 years. At PC diagnosis, the median fasting blood glucose level was 173.5 mg/dL, and the median HbA1c level was 8.2%. The median CEA and CA19-9 levels were 5.0 ng/mL and 207.8 U/mL, respectively.

### 3.3. Comparison of Clinical Characteristics Between PC Patients with NODM and Those with LSDM

At the time of PC diagnosis, 549 patients (24.0%) had NODM and 1742 patients (76.0%) had LSDM. Patients with NODM were significantly younger at PC diagnosis than those with LSDM (70.4 vs. 73.6 years, *p* < 0.001) (Table 2). The mean age at DM diagnosis was substantially higher in patients with NODM than in those with LSDM (69.7 vs. 57.7 years, *p* < 0.001). As expected, the mean interval between DM and PC diagnosis was markedly shorter in the NODM group than in the LSDM group (0.7 vs. 15.5 years, *p* < 0.001).

Proportion of patients with alcohol drinking history was higher in patients with NODM than in those with LSDM (52.7% vs. 45.5%; *p* = 0.004). No significant differences were observed between the NODM and LSDM groups in sex, BMI, history of pancreatic disease, family history of PC, or smoking history. The median fasting blood glucose level at PC diagnosis was lower in patients with NODM than in those with LSDM (159 vs. 179 mg/dL, *p* < 0.001), whereas median HbA1c levels were not statistically different (8.0% vs. 8.3%, *p* = 0.073).

The overall clinical stage distribution did not differ significantly between the NODM and LSDM groups. Stage 0/IA disease was present in 8.9% of patients with NODM and 9.9% of those with LSDM (*p* = 0.53), whereas stage IV disease was present in 33.4% and 37.1%, respectively (*p* = 0.12).

In addition, we performed sensitivity analyses using alternative definitions of NODM and LSDM, with the cutoff set at 1 or 2 years before PC diagnosis. Overall, similar results were observed when the cutoff was set at 1 or 2 years before PC diagnosis (Supplementary Tables S1 and S2).

### 3.4. Primary Diagnostic Triggers for PC

Information regarding the primary diagnostic trigger was available for 2274 patients. PC was diagnosed because of symptoms in 781 patients (34.3%), whereas 1493 patients (65.7%) were diagnosed through non-symptom-based triggers (Table 3). Among the non-symptom-based triggers, worsening LSDM accounted for 24.4%, abnormal imaging findings for 21.4%, abnormal laboratory findings for 15.0%, NODM for 2.8%, and follow-up of known pancreatic disease for 2.2%. Among patients with detailed symptom information, abdominal or back pain was the most common symptom (61.5%), followed by jaundice (30.7%) and body weight loss (16.9%), with some patients reporting multiple symptoms.

The stage distribution also differed substantially by diagnostic trigger. Among patients whose PC was diagnosed because of symptoms, stage 0/IA disease was present in 29 of 741 patients (3.9%), whereas stage IV disease was present in 367 patients (49.5%). In contrast, among patients diagnosed through diabetes-related changes, stage 0/IA disease was present in 64 of 583 patients (11.0%), whereas stage IV disease was present in 152 patients (26.1%). Stage 0/IA disease was more frequent, and stage IV disease was less frequent in patients diagnosed through diabetes-related changes (both *p* < 0.001). The proportion of stage 0 disease did not differ between cases with symptom-based detection and those with diabetes-related detection (0.4% vs. 1.0%; *p* = 0.19).

In multivariable logistic regression analysis, diabetes-related detection was independently associated with stage 0/IA disease, with an adjusted OR of 2.87 (95% CI, 1.72–4.81; *p* < 0.001) after adjustment for age, sex, BMI, pancreatic disease history, family history, smoking, and alcohol consumption (Table 4). A history of pancreatic disease was also independently associated with stage 0/IA PC (adjusted OR, 2.24; 95% CI, 1.40–3.57; *p* < 0.001). The results were essentially unchanged when institution was included as a random intercept in a mixed-effects logistic regression model (adjusted OR, 2.89; 95% CI, 1.72–4.85; *p* < 0.001) (Supplementary Table S3).

Abnormal imaging findings and follow-up of pancreatic diseases represented additional important non-symptom-based routes to diagnosis and were associated with particularly high proportions of stage 0/IA disease (17.1% and 23.4%, respectively).

Patients diagnosed because of symptoms were significantly younger at PC diagnosis than those diagnosed through non-symptom-based triggers (71.9 vs. 73.4 years, *p* < 0.001) (Table 5). The median CEA and CA19-9 levels at PC diagnosis were also significantly higher in the symptom-based group than in the non-symptom-based group (5.6 vs. 4.8 ng/mL, *p* < 0.001; and 422.9 vs. 168.0 U/mL, *p* < 0.001, respectively). These findings were consistent with the more advanced disease stage observed among symptomatic patients. Indeed, the proportion of stage 0/IA disease was substantially lower in the symptom-based group than in the non-symptom-based group (3.9% vs. 12.6%, *p* < 0.001), whereas stage IV disease was more frequent in the symptom-based group (49.5% vs. 29.3%, *p* < 0.001). A history of pancreatic disease was less common in the symptom-based group (9.2% vs. 12.5%, *p* = 0.02). No significant differences were observed in sex, BMI, family history of PC, or smoking history.

### 3.5. Interval Between Worsening of Diabetes and PC Diagnosis

Among 1033 patients with worsening LSDM for whom the interval to PC diagnosis was available, 222 patients (21.5%) were diagnosed with PC within 1 month of worsening diabetes. A further 369 patients (35.7%) were diagnosed between 1 and less than 3 months, 229 patients (22.2%) between 3 and less than 6 months, and 144 patients (13.9%) between 6 and less than 12 months. Only 69 patients (6.7%) were diagnosed more than 1 year after worsening of diabetes. Overall, 57.2% of patients with worsening diabetes were diagnosed with PC within 3 months, and 79.4% within 6 months.

### 3.6. Body Weight Changes Preceding PC Diagnosis

Body weight data at PC diagnosis and 1 year before diagnosis were available for 513 patients. A decrease in body weight of more than 10% between these two time points was observed in 114 patients (22.2%) (Table 6). Weight reductions of 7.5–9.9%, 5.0–7.4%, and 2.5–4.9% occurred in 12.7%, 13.6%, and 18.3% of patients, respectively. Overall, 66.9% of the patients with available data experienced a weight reduction of at least 2.5%. The proportion of patients with more than 10% weight loss was similar between the NODM and LSDM groups (25.0% and 21.8%, respectively; *p* = 0.56). Clinical characteristics differed between patients with and without available body-weight data in several respects, including age, diabetes duration, and history of pancreatic disease (Supplementary Table S4). In particular, the proportion of patients with NODM was substantially higher among those with missing body-weight data. This may partly reflect the fact that many patients with NODM had not yet been diagnosed with diabetes 1 year before PC diagnosis, resulting in less frequent availability of body-weight measurements at that time. These differences suggest that patients with available body-weight data may not be fully representative of the overall study population.

### 3.7. Changes in HbA1c Preceding PC Diagnosis

HbA1c values at PC diagnosis and 1 year before diagnosis were available for 778 patients. An HbA1c increase of ≥5.0 percentage points was observed in 37 patients (4.8%), while an increase of ≥1.0 percentage point was observed in 361 (46.4%) (Table 7). An HbA1c increase of ≥5.0 percentage points was more frequent in patients with NODM than in those with LSDM (15.7% vs. 3.1%, *p* < 0.001). As in the case of body weight data, clinical characteristics also differed between patients with and without available longitudinal HbA1c data (Supplementary Table S5), and informative missingness therefore cannot be excluded.

## 4. Discussion

In this nationwide, multicenter survey conducted through JDS-certified educational facilities, we examined how PC was diagnosed among patients receiving specialist diabetes care, with particular attention to diabetes-related changes beyond NODM. Approximately three-quarters of patients with PC had LSDM. Many of these patients had worsening diabetes documented before PC diagnosis, and diabetes-related diagnostic triggers were associated with more stage 0/IA disease and less stage IV disease than symptom-based presentation. Among patients with worsening LSDM, more than half were diagnosed with PC within 3 months and nearly 80% within 6 months. Substantial weight loss and increases in HbA1c were also frequently observed among patients with available longitudinal data. These findings describe clinical patterns among patients who ultimately developed PC; they do not establish that deterioration in glycemic control prospectively predicts PC or improves early cancer detection.

Previous studies have focused largely on NODM as a potential clinical clue to PC [15,16,17,18,19]. However, NODM represented only a subset of patients in our cohort, whereas approximately three-quarters had LSDM. Worsening glycemic control in pre-existing diabetes has also been reported to precede PC diagnosis [34,35]. Our findings further characterize such changes in specialist diabetes practice. However, because our study did not include a control group of patients with diabetes without PC, we cannot determine whether these changes distinguish cancer-associated deterioration from the ordinary progression of diabetes.

The median facility-level frequency of PC encountered in specialist diabetes practice was 2.0 per 1000 patients with DM. This value describes the frequency with which PC was encountered in specialist diabetes practice and should not be interpreted as a population-based incidence or prevalence estimate. Previous population-based studies have consistently shown that patients with DM have an increased risk of PC [36,37]. Our nationwide survey complements these epidemiological data by describing how PC was encountered in specialist diabetes care and providing context for the subsequent analyses of diagnostic triggers and diabetes-related changes.

The short interval between worsening LSDM and PC diagnosis was notable, with more than half of these patients diagnosed within 3 months and nearly 80% within 6 months of documented diabetes deterioration. This temporal clustering may reflect both PC-related metabolic changes and clinical practice. PC can cause metabolic abnormalities through impaired insulin secretion, insulin resistance, systemic inflammation, and other tumor-associated mechanisms, and longitudinal studies have demonstrated progressive deterioration in glucose metabolism before PC diagnosis [9,14,21,22]. Patients with unexpected worsening of glycemic control may also undergo more intensive laboratory testing and imaging, which could shorten the interval to cancer diagnosis. Accordingly, this temporal association should not be interpreted as establishing a prospective detection signal.

The association between the diagnostic trigger and disease stage provides an additional clinically important finding. Patients whose PC was detected through diabetes-related changes had substantially more stage 0/IA disease and less stage IV disease than those diagnosed after symptom development. Diabetes-related detection remained independently associated with stage 0/IA PC after multivariable adjustment, suggesting that diabetes-related changes may represent a clinically relevant route to PC diagnosis before symptom development. Similarly, patients diagnosed through non-symptomatic triggers had earlier disease than those diagnosed after symptom presentation. Takikawa et al. reported that asymptomatic PC was characterized by smaller tumors, earlier stage, and higher resectability than symptomatic PC, with diabetes onset or exacerbation accounting for an important proportion of asymptomatic detections [38]. In another Japanese multicenter study, only 25% of stage 0/IA PC cases were symptomatic, highlighting the importance of abnormalities detected during examinations or follow-up for other diseases [32]. Our nationwide study extends these observations to specialist diabetes care and suggests that diabetes management itself may provide an opportunity for earlier recognition of PC. These findings suggest that clinical attention should extend beyond NODM to unexpected changes in established diabetes.

The differences according to symptom status provide additional context. Patients with symptomatic PC were younger and had higher serum CEA and CA19-9 levels than those without symptoms. The higher tumor marker levels may reflect greater tumor burden or more advanced disease at the time of symptom development, although the cross-sectional nature of this comparison precludes determining the temporal relationship. Although CA19-9 and CEA may support the diagnosis of PC, their limited sensitivity, particularly in early-stage disease, and limited specificity preclude their use as stand-alone biomarkers for early detection or population screening [39,40]. These findings further underscore that differences between symptom-based and non-symptom-based diagnostic routes may reflect when and how intensively patients are evaluated rather than a direct effect of metabolic deterioration on cancer stage.

Our findings have important clinical implications, but they should not be interpreted as supporting routine pancreatic imaging for every patient with worsening glycemic control. Because DM is highly prevalent whereas the absolute risk of PC remains low, indiscriminate imaging would likely lead to substantial numbers of unnecessary examinations and incidental findings. Current recommendations generally restrict pancreatic surveillance to selected high-risk populations rather than average-risk individuals [7,8]. The present study does not establish a clinically actionable threshold for pancreatic investigation based on HbA1c increase, weight loss, treatment escalation, or any combination of these features.

Risk stratification tools may help identify patients who warrant further evaluation. The ENDPAC model, which incorporates age at diabetes onset, weight change, and change in blood glucose, was developed to identify patients with NODM at increased risk of PC [18], and subsequent studies have supported its potential utility [41]. However, its application has primarily focused on NODM. Our findings suggest that a similar trajectory-based approach could be extended to patients with LSDM. Future models may incorporate the magnitude and rate of HbA1c increase, weight loss, diabetes duration, age, treatment escalation, and established PC risk factors. A particularly relevant phenotype may be rapid and unexplained deterioration in glycemic control accompanied by weight loss, regardless of diabetes duration. Combining such metabolic trajectories with selected imaging or circulating biomarkers may further enrich the population in whom pancreatic imaging is warranted [42]. Endoscopic ultrasound, including endoscopic ultrasound-guided tissue acquisition when appropriate, provides high-resolution assessment and tissue diagnosis of pancreatic lesions [43,44]. However, the present study does not establish which patients with diabetes deterioration should undergo such imaging.

This study has several limitations. First, the first-stage response rate was relatively low, and participating institutions were JDS-certified educational facilities representing specialist diabetes care; therefore, selection bias is possible and the findings may not be generalizable to community-based diabetes practice. Second, the retrospective design meant that the timing and definition of worsening diabetes were based on routine clinical assessment rather than prespecified quantitative criteria, and diagnostic intensity may have differed among institutions and patients. Third, longitudinal body-weight and HbA1c data were available only for subsets of the cohort, and informative missingness cannot be excluded. Fourth, because the cohort consisted exclusively of patients who ultimately developed PC and lacked a control group of patients with diabetes without PC, the study cannot determine the predictive value, specificity, positive predictive value, or absolute risk associated with worsening glycemic control or weight loss. Prospective studies beginning at the time of diabetes deterioration are therefore required to determine the absolute risk associated with specific metabolic trajectories and to establish clinically meaningful thresholds for further investigation [23]. Finally, the association between diabetes-related detection and stage may partly reflect detection-mode bias and differences in surveillance intensity and does not establish improved detection, resectability, or survival. Despite these limitations, this nationwide multicenter survey provides a unique description of PC encountered in specialist diabetes practice and includes more than 2000 patients with detailed information on diagnostic triggers, diabetes duration, symptoms, disease stage, and metabolic changes.

## 5. Conclusions

Our findings show that most patients with PC encountered in specialist diabetes practice had long-standing rather than new-onset diabetes. Among those who ultimately developed PC, deterioration in glycemic control and weight loss were frequently observed before PC diagnosis, and diabetes-related diagnostic triggers were associated with less advanced disease than symptom-based presentation. These findings should be regarded as hypothesis-generating, and prospective studies are needed to determine whether specific diabetes trajectories can identify patients at sufficiently increased risk to warrant targeted pancreatic evaluation.

## Figures and Tables

**Table 1:** Clinical characteristics of the 2291 patients with PC.

Variables	Total (n = 2291)
Sex, male, n (%)	1434 (62.6)
BMI, kg/m^2^, mean (SD)	22.6 (3.7) ^a^
History of pancreatic diseases	253 (11.4) ^b^
Family history of PC	138 (8.1) ^c^
Alcohol drinking history, ever, n (%)	1000 (47.2) ^d^
Cigarette smoking history, ever, n (%)	1155 (53.4) ^e^
Age at diagnosis of PC, years, mean (SD)	72.8 (9.2) ^f^
Age group, n (%)	
<60 years	180 (7.9)
60−69 years	577 (25.3)
≥70 years	1525 (66.8)
Age at diagnosis of DM, years, mean (SD)	60.9 (12.6) ^g^
New-onset DM (≤3 years), n (%)	549 (24.0)
Long-standing DM (>3 years), n (%)	1742 (76.0)
Interval between DM and PC diagnoses, years, mean (SD)	11.5 (10.9) ^h^
Detailed interval between DM and PC diagnoses, n (%) ^h^	
Within 1 month	253 (12.6)
>1 to 6 months	77 (3.8)
>6 to 12 months	48 (2.4)
>1 to 2 years	73 (3.6)
>2 to 3 years	98 (4.9)
>3 to 5 years	101 (5.0)
>5 to 10 years	391 (19.4)
>10 to 20 years	521 (25.9)
>20 years	449 (22.3)
Clinical stage, n (%) ^i^	
0	28 (1.3)
IA	179 (8.3)
0/IA	207 (9.6)
IB	169 (7.9)
I	348 (16.2)
II	719 (33.4)
III	277 (12.9)
IV	779 (36.2)
Laboratory findings at PC diagnosis	
FBG, mg/dL, median (IQR)	173.5 (133−244) ^j^
HbA1c, %, median (IQR)	8.2 (7.1−9.8) ^k^
CEA, ng/mL, median (IQR)	5.0 (3.0−10.5) ^l^
CA19-9, U/mL, median (IQR)	207.8 (36.0−1251.3) ^m^

Data from ^a^ 2145, ^b^ 2227, ^c^ 1704, ^d^ 2117, ^e^ 2161, ^f^ 2282, ^g^ 2014, ^h^ 2011, ^i^ 2151, ^j^ 1746, ^k^ 2147, ^l^ 2146, and ^m^ 2163 patients. BMI, body mass index; CA19-9, carbohydrate antigen 19-9; CEA, carcinoembryonic antigen; DM, diabetes mellitus; FBG, fasting blood glucose; HbA1c, glycated hemoglobin; IQR, interquartile range; PC, pancreatic cancer; SD, standard deviation.

**Table 2:** Comparison of clinical characteristics between patients with NODM and LSDM.

Variables	NODM n = 549 (24.0%)	LSDM n = 1742 (76.0%)	*p* Value
Sex, male, n (%)	335 (61.0)	1099 (63.1)	0.38
BMI, kg/m^2^, mean (SD)	22.6 (3.5) ^a^	22.7 (3.8) ^b^	0.78
History of pancreatic diseases	52 (9.7) ^c^	201 (11.9) ^d^	0.15
Family history of PC	38 (8.7) ^e^	100 (7.9) ^f^	0.57
Alcohol drinking history, ever, n (%)	274 (52.7) ^g^	726 (45.5) ^h^	0.004
Cigarette smoking history, ever, n (%)	279 (53.1) ^i^	876 (53.5) ^j^	0.87
Age at PC diagnosis, mean (SD)	70.4 (9.9)	73.6 (8.8) ^k^	<0.001
Age at DM diagnosis, mean (SD)	69.7 (10.0)	57.7 (11.8) ^l^	<0.001
Interval between DM and PC, mean (SD)	0.7 (0.9)	15.5 (10.2) ^m^	<0.001
Laboratory findings at PC diagnosis			
FBG, mg/dL, median (IQR)	159 (127–219.5) ^n^	179 (136–248.25) ^o^	<0.001
HbA1c, %, median (IQR)	8.0 (6.9–10) ^p^	8.3 (7.2–9.8) ^q^	0.073
CEA, ng/mL, median (IQR)	4.9 (3–10.025) ^r^	5 (3–10.6) ^s^	0.28
CA19-9, U/mL, median (IQR)	172.6 (32–983) ^t^	223.4 (38.9–1387.85) ^u^	0.053
Primary trigger for PC diagnosis, n (%)	n = 544	n = 1730	
Symptom	198 (36.4)	583 (33.7)	0.25
Worsening of pre-existing DM	83 (15.3)	471 (27.2)	<0.001
New-onset DM	63 (11.6)	0 (0)	<0.001
Abnormal imaging findings	111 (20.4)	375 (21.7)	0.53
Abnormal laboratory findings	78 (14.3)	262 (15.1)	0.65
Follow-up of pancreatic disease	11 (2.0)	39 (2.3)	0.75
Clinical stage, n (%)	n = 527	n = 1624	
0	6 (1.1)	22 (1.4)	0.70
IA	41 (7.8)	138 (8.5)	0.60
0/IA	47 (8.9)	160 (9.9)	0.53
IB	42 (8.0)	127 (7.8)	0.91
I	83 (15.8)	265 (16.3)	0.76
II	194 (36.8)	525 (32.3)	0.058
III	68 (12.9)	209 (12.9)	0.98
IV	176 (33.4)	603 (37.1)	0.12

Data from ^a^ 514, ^b^ 1631, ^c^ 538, ^d^ 1689, ^e^ 435, ^f^ 1269, ^g^ 520, ^h^ 1597, ^i^ 525, ^j^ 1636, ^k^ 1733, ^l^ 1465, ^m^ 1462, ^n^ 448, ^o^ 1298, ^p^ 519, ^q^ 1628, ^r^ 518, ^s^ 1616, ^t^ 523, and ^u^ 1640 patients. BMI, body mass index; CA19-9, carbohydrate antigen 19-9; CEA, carcinoembryonic antigen; DM, diabetes mellitus; FBG, fasting blood glucose; HbA1c, glycated hemoglobin; IQR, interquartile range; LSDM, long-standing DM; NODM, new-onset DM; PC, pancreatic cancer; SD, standard deviation.

**Table 3:** Distribution of clinical stages according to the primary trigger for PC diagnosis.

	Primary Trigger for PC Diagnosis, n (%)							
Clinical Stage	Symptom	DM-Related			Abnormal Imaging Findings	Abnormal Laboratory Findings	Follow-Up of Pancreatic Diseases	Total
		All DM- Related	Worsening LSDM	NODM				
0	3 (0.4)	6 (1.0)	5 (1.0)	1 (1.7)	8 (1.8)	4 (1.2)	7 (14.9)	28
IA	26 (3.5)	58 (9.9)	54 (10.3)	4 (6.7)	68 (15.3)	21 (6.5)	4 (8.5)	177
0/IA	29 (3.9)	64 (11.0)	59 (11.3)	5 (8.3)	76 (17.1)	25 (7.7)	11 (23.4)	205
IB	37 (5.0)	47 (8.1)	43 (8.2)	4 (6.7)	46 (10.3)	26 (8.0)	12 (25.5)	168
I	63 (8.5)	105 (18.0)	97 (18.5)	8 (13.3)	114 (25.6)	47 (14.5)	16 (34.0)	345
II	190 (25.6)	244 (41.9)	215 (41.1)	29 (48.3)	150 (33.7)	117 (36.1)	15 (31.9)	716
III	118 (15.9)	76 (13.0)	67 (12.8)	9 (15.0)	37 (8.3)	41 (12.7)	3 (6.4)	275
IV	367 (49.5)	152 (26.1)	139 (26.6)	13 (21.7)	136 (30.6)	115 (35.5)	6 (12.8)	776
Subtotal	741 (100)	583 (100)	523 (100)	60 (100)	445 (100)	324 (100)	47 (100)	2140
Unknown, n	40	34	31	3	41	16	3	
Total	781 (34.3)	617 (27.1)	554 (24.4)	63 (2.8)	486 (21.4)	340 (15.0)	50 (2.2)	

Primary trigger for PC diagnosis was unknown in 2 patients with stage IA, 1 with stage IB, 3 with stage II, 2 with stage III, and 3 with stage IV. DM, diabetes mellitus; LSDM, long-standing DM; NODM, new-onset DM; PC, pancreatic cancer.

**Table 4:** Factors associated with stage 0/IA PC in a multivariable analysis.

Variables	Adjusted OR	95% CI	*p* Value
Diabetes-related vs. symptom-based detection	2.87	1.72–4.81	<0.001
Age, per year	1.00	0.98–1.02	0.90
Male sex (vs. female)	0.72	0.47–1.12	0.15
Body mass index, per kg/m^2^	1.00	0.96–1.05	0.92
History of pancreatic diseases	2.24	1.40–3.57	<0.001
Family history of pancreatic cancer	1.74	1.01–3.01	0.046
Alcohol drinking history, ever (vs. never)	0.80	0.54–1.18	0.27
Cigarette smoking history, ever (vs. never)	1.34	0.87–2.05	0.18

Stage IB–IV served as the reference outcome. CI, confidence interval; OR, odds ratio.

**Table 5:** Comparison of clinical characteristics of patients with PC diagnosed because of symptoms and those diagnosed through non-symptom-based triggers.

Variables	Symptom-Based n = 781 (34.3%)	Non-Symptom-Based n = 1493 (65.7%)	*p* Value
Sex, male, n (%)	489 (62.6)	935 (62.6)	0.99
BMI, kg/m^2^, mean (SD)	22.5 (3.8) ^a^	22.7 (3.8) ^b^	0.13
History of pancreatic diseases	70 (9.2) ^c^	182 (12.5) ^d^	0.02
Family history of PC	47 (8.5) ^e^	89 (7.8) ^f^	0.62
Alcohol drinking history, ever, n (%)	341 (48.1) ^g^	655 (46.9) ^h^	0.60
Cigarette smoking history, ever, n (%)	387 (54.0) ^i^	762 (53.2) ^j^	0.75
Age at PC diagnosis, mean (SD)	71.9 (9.7) ^k^	73.4 (8.9) ^l^	<0.001
Age at DM diagnosis, mean (SD)	60.1 (12.7) ^m^	61.4 (12.5) ^n^	0.028
Interval between DM and PC, mean (SD)	11.2 (10.8) ^o^	11.7 (11.0) ^p^	0.43
Laboratory findings at PC diagnosis, median (IQR)			
FBG, mg/dL, median (IQR)	165 (131–230) ^q^	177 (134–249) ^r^	0.046
HbA1c, %, median (IQR)	7.8 (6.9–9.1) ^s^	8.5 (7.3–10.1) ^t^	<0.001
CEA, ng/mL, median (IQR)	5.6 (3.1–15.25) ^u^	4.8 (3–9.2) ^v^	<0.001
CA19-9, U/mL, median (IQR)	422.9 (48–3134) ^w^	168.0 (33.9–801) ^x^	<0.001
Clinical stage, n (%)	n = 741	n = 1398	
0	3 (0.4)	24 (1.7)	0.008
IA	26 (3.5)	152 (10.9)	<0.001
0/IA	29 (3.9)	176 (12.6)	<0.001
IB	37 (5.0)	131 (9.4)	<0.001
I	63 (8.5)	283 (20.2)	<0.001
II	190 (25.6)	526 (37.6)	<0.001
III	118 (15.9)	156 (11.2)	0.002
IV	367 (49.5)	409 (29.3)	<0.001

Data from ^a^ 740, ^b^ 1399, ^c^ 761, ^d^ 1454, ^e^ 553, ^f^ 1141, ^g^ 709, ^h^ 1397, ^i^ 717, ^j^ 1431, ^k^ 779, ^l^ 1486, ^m^ 680, ^n^ 1319, ^o^ 679, ^p^ 1317, ^q^ 587, ^r^ 1153, ^s^ 731, ^t^ 1408, ^u^ 741, ^v^ 1384, ^w^ 747, and ^x^ 1407 patients. Primary trigger for PC diagnosis was unknown in 17 patients. DM, diabetes mellitus, PC, pancreatic cancer.

**Table 6:** Change in body weight from 1 year before to PC diagnosis.

Body Weight Change	Total, n (%)	NODM, n (%)	LSDM, n (%)
≤−10.0%	114 (22.2)	17 (25.0)	97 (21.8)
−9.9% to −7.5%	65 (12.7)	9 (13.2)	56 (12.6)
−7.4% to −5.0%	70 (13.6)	7 (10.3)	63 (14.2)
−4.9% to −2.5%	94 (18.3)	11 (16.2)	83 (18.7)
−2.4% to +2.4%	122 (23.8)	20 (29.4)	102 (22.9)
+2.5% to +4.9%	30 (5.8)	3 (4.4)	27 (6.1)
+5.0% to +7.4%	10 (1.9)	1 (1.5)	9 (2.0)
+7.5% to +9.9%	5 (1.0)	0 (0.0)	5 (1.1)
≥+10.0%	3 (0.6)	0 (0.0)	3 (0.7)
Total	513 (100)	68 (100)	445 (100)

Body weight change was rounded to one decimal place before categorization. DM, diabetes mellitus; NODM, new-onset DM; LSDM, long-standing DM; PC, pancreatic cancer.

**Table 7:** Change in HbA1c from 1 year before to PC diagnosis.

HbA1c Change	Total, n (%)	NODM, n (%)	LSDM, n (%)
≥+5.0%	37 (4.8)	16 (15.7)	21 (3.1)
+4.0% to +4.9%	25 (3.2)	6 (5.9)	19 (2.8)
+3.0% to +3.9%	59 (7.6)	5 (4.9)	54 (8.0)
+2.0% to +2.9%	84 (10.8)	10 (9.8)	74 (10.9)
+1.0% to +1.9%	156 (20.1)	25 (24.5)	131 (19.4)
+0.5% to +0.9%	103 (13.2)	10 (9.8)	93 (13.8)
+0.3% to +0.4%	43 (5.5)	4 (3.9)	39 (5.8)
−0.2% to +0.2%	124 (15.9)	12 (11.8)	112 (16.6)
−0.3% to −0.4%	37 (4.8)	4 (3.9)	33 (4.9)
−0.5% to −0.9%	48 (6.2)	7 (6.9)	41 (6.1)
−1.0% to −1.9%	35 (4.5)	0 (0)	35 (5.2)
−2.0% to −2.9%	17 (2.2)	3 (2.9)	14 (2.1)
≤−3.0%	10 (1.3)	0 (0)	10 (1.5)
Total	778 (100)	102 (100)	676 (100)

DM, diabetes mellitus; HbA1c, glycated hemoglobin A1c; LSDM, long-standing DM; NODM, new-onset DM; PC, pancreatic cancer.

## Data Availability

The datasets generated and/or analyzed during the current study are available from the corresponding author on reasonable request.

## Supplementary Materials

The following supporting information can be downloaded at https://www.mdpi.com/article/10.3390/cancers18182995/s1, Table S1: Comparison of clinical characteristics between patients with NODM and LSDM using a 1-year cutoff before PC diagnosis; Table S2: Comparison of clinical characteristics between patients with NODM and LSDM using a 2-year cutoff before PC diagnosis; Table S3: Factors associated with stage 0/IA PC in a mixed-effects logistic regression analysis; Table S4: Comparison of patient characteristics according to the availability of body-weight data; Table S5: Comparison of patient characteristics according to the availability of HbA1c data.

## Abbreviations

The following abbreviations are used in this manuscript:

BMI	body mass index
CI	confidence interval
DM	diabetes mellitus
ENDPAC	Enriching New-Onset Diabetes for Pancreatic Cancer
HbA1c	glycated hemoglobin A1c
IQR	interquartile range
JDS	Japan Diabetes Society
NODM	new-onset DM
OR	Odds ratio
LSDM	long-standing DM
PC	pancreatic cancer
SD	standard deviation
