# Supplementary material for: Diabetes Trajectory and Pancreatic Cancer Detection in Patients with Diabetes: A Nationwide Survey Through Japan Diabetes Society-Certified Facilities
Source: Cancers (Basel). 2026 Sep 16;18(18):2995. doi: 10.3390/cancers18182995 (PMC13604488; doi:10.3390/cancers18182995)
Supplement: Supplementary file 1 [file cancers-18-02995-s001.zip › cancers-4534730-supplementary.pdf]

Supplementary Table S1. Comparison of clinical characteristics between patients with NODM and LSDM using a 1-year cutoff before PC diagnosis.

**Variables**	**NODM** n = 378 (16.5%)	**LSDM** n = 1913 (83.5%)	***p* Value**
Sex, male, n (%)	213 (56.4)	1221 (63.8)	0.006
BMI, kg/m^2, mean (SD)	22.6 (3.6) [a]	22.6 (3.8) [b]	0.85
History of pancreatic diseases	32 (8.6) [c]	221 (11.9) [d]	0.07
Family history of PC	28 (9.2) [e]	110 (7.9) [f]	0.43
Alcohol drinking history, ever, n (%)	184 (51.5) [g]	816 (46.4) [h]	0.07
Cigarette smoking history, ever, n (%)	179 (49.5) [i]	976 (54.3) [j]	0.09
Age at PC diagnosis, mean (SD)	70.3 (9.7)	73.3 (9.0) [k]	< 0.001
Age at DM diagnosis, mean (SD)	70.1 (9.7)	58.8 (12.2) [l]	< 0.001
Interval between DM and PC, mean (SD)	0.2 (0.4)	14.1 (10.5) [m]	< 0.001
Laboratory findings at PC diagnosis			
FBG, mg/dL, median (IQR)	160 (128–218) [n]	178 (134–247) [o]	0.006
HbA1c, %, median (IQR)	7.9 (6.9–10.5) [p]	8.3 (7.2–9.8) [q]	0.12
CEA, ng/mL, median (IQR)	4.9 (3.0–10.5) [r]	5.0 (3.0–10.5) [s]	0.77
CA19-9, U/mL, median (IQR)	180.1 (35.2–1031) [t]	214.2 (37.2–1308) [u]	0.40
Primary trigger for PC diagnosis, n (%)	n = 375	n = 1899	
Symptom	146 (38.9)	635 (33.4)	0.04
Worsening of pre-existing DM	35 (9.3)	519 (27.3)	< 0.001
New-onset DM	62 (16.5)	1 (0.05)	< 0.001
Abnormal imaging findings	78 (20.8)	408 (21.5)	0.77
Abnormal laboratory findings	49 (13.1)	291 (15.3)	0.26
Follow-up of pancreatic disease	5 (1.3)	45 (2.4)	0.21
Clinical stage, n (%)	n = 360	n = 1791	
0	3 (0.8)	25 (1.4)	0.39
IA	30 (8.3)	149 (8.3)	0.99
0/IA	33 (9.2)	174 (9.7)	0.75
IB	24 (6.7)	145 (8.1)	0.36
I	54 (15.0)	294 (16.4)	0.51
II	143 (39.7)	576 (32.2)	0.006
III	45 (12.5)	232 (13.0)	0.81
IV	115 (31.9)	664 (37.1)	0.06

Data from [a] 351, [b] 1794, [c] 370, [d] 1857, [e] 304, [f] 1400, [g] 357, [h] 1760, [i] 362, [j] 1799, [k] 1904, [l] 1636, [m] 1633, [n] 314, [o] 1431, [p] 358, [q] 1789, [r] 353, [s] 1781, [t] 357, and [u] 1807 patients.

BMI, body mass index; CA19-9, carbohydrate antigen 19-9; CEA, carcinoembryonic antigen; DM, diabetes mellitus; FBG, fasting blood glucose; HbA1c, glycated hemoglobin; IQR, interquartile range; LSDM, long-standing DM; NODM, new-onset DM; PC, pancreatic cancer; SD, standard deviation.

Supplementary Table S2. Comparison of clinical characteristics between patients with NODM and LSDM using a 2-year cutoff before PC diagnosis.

**Variables**	**NODM** **n = 451 (19.7%)**	**LSDM** **n = 1840 (80.3%)**	***p* Value**
Sex, male, n (%)	262 (58.1)	1172 (63.7)	0.03
BMI, kg/m^2, mean (SD)	22.6 (3.6) [a]	22.6 (3.8) [b]	0.80
History of pancreatic diseases	39 (8.8) [c]	214 (12.0) [d]	0.06
Family history of PC	32 (9.0) [e]	106 (7.9) [f]	0.45
Alcohol drinking history, ever, n (%)	223 (52.4) [g]	777 (46.0) [h]	0.02
Cigarette smoking history, ever, n (%)	220 (50.9) [i]	935 (54.1) [j]	0.24
Age at PC diagnosis, mean (SD)	70.4 (9.8)	73.4 (8.9) [k]	< 0.001
Age at DM diagnosis, mean (SD)	70.0 (9.8)	58.3 (12.0) [l]	< 0.001
Interval between DM and PC, mean (SD)	0.3 (0.6)	14.7 (10.4) [m]	< 0.001
Laboratory findings at PC diagnosis			
FBG, mg/dL, median (IQR)	160 (128–226.3) [n]	178 (135–247) [o]	0.003
HbA1c, %, median (IQR)	8.0 (6.9–10.4) [p]	8.3 (7.2–9.8) [q]	0.16
CEA, ng/mL, median (IQR)	5.0 (3.0–10.4) [r]	5.0 (3.0–10.5) [s]	0.64
CA19-9, U/mL, median (IQR)	175.9 (35–971) [t]	219.0 (37.6–1330.5) [u]	0.18
Primary trigger for PC diagnosis, n (%)	n = 447	n = 1827	
Symptom	167 (37.4)	614 (33.6)	0.13
Worsening of pre-existing DM	55 (12.3)	499 (27.3)	< 0.001
New-onset DM	63 (14.1)	0 (0)	< 0.001
Abnormal imaging findings	97 (21.7)	389 (21.3)	0.85
Abnormal laboratory findings	58 (13.0)	282 (15.4)	0.19
Follow-up of pancreatic disease	7 (1.6)	43 (2.4)	0.31
Clinical stage, n (%)	n = 431	n = 1720	
0	4 (0.9)	24 (1.4)	0.44
IA	35 (8.1)	144 (8.4)	0.87
0/IA	39 (9.1)	168 (9.8)	0.65
IB	34 (7.9)	135 (7.9)	0.98
I	69 (16.0)	279 (16.2)	0.92
II	157 (36.4)	562 (32.7)	0.14
III	56 (13.0)	221 (12.9)	0.94
IV	145 (33.6)	634 (36.9)	0.21

Data from [a] 420, [b] 1725, [c] 441, [d] 1786, [e] 357, [f] 1347, [g] 426, [h] 1691, [i] 432, [j] 1729, [k] 1831, [l] 1563, [m] 1560, [n] 368, [o] 1377, [p] 424, [q] 1723, [r] 422, [s] 1712, [t] 427, and [u] 1737 patients.

BMI, body mass index; CA19-9, carbohydrate antigen 19-9; CEA, carcinoembryonic antigen; DM, diabetes mellitus; FBG, fasting blood glucose; HbA1c, glycated hemoglobin; IQR, interquartile range; LSDM, long-standing DM; NODM, new-onset DM; PC, pancreatic cancer; SD, standard deviation

Supplementary Table S3. Factors associated with stage 0/IA PC in a mixed-effects logistic regression analysis.

**Variables**	**Adjusted OR**	**95% CI**	***p* Value**
Diabetes-related vs. symptom-based detection	2.89	1.72–4.85	< 0.001
Age, per year	1.00	0.98–1.02	0.76
Male sex (vs. female)	0.72	0.46–1.12	0.15
Body mass index, per kg/m^2	1.00	0.95–1.05	0.98
History of pancreatic diseases	2.31	1.43–3.72	< 0.001
Family history of pancreatic cancer	1.73	0.99–3.01	0.052
Alcohol drinking history, ever (vs. never)	0.79	0.53–1.18	0.25
Cigarette smoking history, ever (vs. never)	1.35	0.87–2.08	0.18

Institution was included as a random intercept. Stage IB–IV served as the reference outcome. CI, confidence interval; OR. Odds ratio; PC, pancreatic cancer.

Supplementary Table S4. Comparison of patient characteristics according to the availability of body-weight data.

Variables	Data Available n = 513	Data Missing n = 1778	*p* Value
Sex, male, n (%)	347 (67.6)	1087 (61.1)	0.007
BMI, kg/m^2, mean (SD)	23.0 (3.9)	22.5 (3.7) [a]	0.03
History of pancreatic diseases, n (%)	84 (16.9)	169 (9.8) [b]	< 0.001
Family history of PC	34 (8.7)	104 (7.9) [c]	0.60
Alcohol drinking history, ever, n (%)	217 (44.6)	783 (48.0) [d]	0.18
Cigarette smoking history, ever, n (%)	273 (55.0)	882 (53.0) [e]	0.42
Age at diagnosis of PC, years, mean (SD)	73.8 (9.1)	72.6 (9.2) [f]	0.007
Age at diagnosis of DM, years, mean (SD)	59.0 (13.4)	61.6 (12.2)	< 0.001
Diabetes duration category, n (%)			< 0.001
New-onset DM (≤ 3 years)	68 (13.3)	481 (27.1)	
Long-standing DM (> 3 years)	445 (86.7)	1297 (73.0)	
Interval between DM and PC diagnoses, years, mean (SD)	14.7 (11.3)	10.5 (10.6)	< 0.001
Detailed interval between DM and PC diagnoses, n (%)	n = 483	n = 1528	
Within 1 month	14 (2.9)	239 (15.6)	< 0.001
> 1 to 6 months	10 (2.1)	67 (4.4)	0.02
> 6 to 12 months	10 (2.1)	38 (2.5)	0.60
> 1 to 2 years	23 (4.8)	50 (3.3)	0.13
> 2 to 3 years	11 (2.3)	87 (5.7)	0.002
> 3 to 5 years	21 (4.3)	80 (5.2)	0.44
> 5 to 10 years	103 (21.3)	288 (18.8)	0.23
> 10 to 20 years	141 (29.2)	380 (24.9)	0.06
> 20 years	150 (31.1)	299 (19.6)	< 0.001
Clinical stage, n (%)	n = 475	n = 1676	
0	9 (1.9)	19 (1.1)	0.20
IA	42 (8.8)	137 (8.2)	0.64
0/IA	51 (10.7)	156 (9.3)	0.35
IB	31 (6.5)	138 (8.2)	0.22
I	73 (15.4)	275 (16.4)	0.59
II	149 (31.4)	570 (34.0)	0.28
III	58 (12.2)	219 (13.1)	0.62
IV	186 (39.2)	593 (35.4)	0.13

BMI, body mass index; DM, diabetes mellitus; PC, pancreatic cancer; SD, standard deviation.

Supplementary Table S5. Comparison of patient characteristics according to the availability of HbA1c data.

Variables	Data Available n = 778	Data Missing n = 1513	*p* Value
Sex, male, n (%)	513 (65.9)	921 (60.9)	0.02
BMI, kg/m^2, mean (SD)	22.9 (3.8)	22.5 (3.7) [a]	0.046
History of pancreatic diseases, n (%)	120 (15.8)	133 (9.1) [b]	< 0.001
Family history of PC, n (%)	47 (8.0)	91 (8.2) [c]	0.87
Alcohol drinking history, ever, n (%)	340 (46.6)	660 (47.6) [d]	0.66
Cigarette smoking history, ever, n (%)	406 (54.4)	749 (53.0) [e]	0.54
Age at diagnosis of PC, years, mean (SD)	73.7 (9.1)	72.4 (9.2) [f]	< 0.001
Age at diagnosis of DM, years, mean (SD)	59.8 (12.9)	61.6 (12.3)	0.005
Diabetes duration category, n (%)			< 0.001
New-onset DM (≤ 3 years), n (%)	102 (13.1)	447 (29.5)	
Long-standing DM (> 3 years), n (%)	676 (86.9)	1066 (70.5)	
Interval between DM and PC diagnoses, years, mean (SD)	13.7 (10.7)	10.3 (10.9)	< 0.001
Detailed interval between DM and PC diagnoses, n (%)	n = 712	n = 1299	
Within 1 month	20 (2.8)	233 (17.9)	< 0.001
> 1 to 6 months	14 (2.0)	63 (4.8)	0.001
> 6 to 12 months	13 (1.8)	35 (2.7)	0.22
> 1 to 2 years	32 (4.5)	41 (3.2)	0.13
> 2 to 3 years	23 (3.2)	75 (5.8)	0.01
> 3 to 5 years	37 (5.2)	64 (4.9)	0.79
> 5 to 10 years	161 (22.6)	230 (17.7)	0.008
> 10 to 20 years	224 (31.5)	297 (22.9)	< 0.001
> 20 years	188 (26.4)	261 (20.1)	0.001
Clinical stage, n (%)	n = 715	n = 1436	
0	10 (1.4)	18 (1.3)	0.78
IA	64 (9.0)	115 (8.0)	0.46
0/IA	74 (10.4)	133 (9.3)	0.42
IB	61 (8.5)	108 (7.5)	0.41
I	125 (17.5)	223 (15.5)	0.25
II	212 (29.7)	507 (35.3)	0.009
III	96 (13.4)	181 (12.6)	0.59
IV	272 (38.0)	507 (35.3)	0.21

BMI, body mass index; DM, diabetes mellitus; PC, pancreatic cancer; SD, standard deviation.
